# High-Flow Nasal Therapy in Acute and Chronic Respiratory Failure: Past, Present, and Future

**DOI:** 10.3390/jcm12072666

**Published:** 2023-04-03

**Authors:** Claudia Crimi, Andrea Cortegiani

**Affiliations:** 1 Department of Clinical and Experimental Medicine, University of Catania, 95123 Catania, Italy; 2 Respiratory Medicine Unit, Policlinico “G. Rodolico-San Marco” University Hospital, 95123 Catania, Italy; 3 Department of Surgical, Oncological and Oral Science (Di.Chir.On.S.), University of Palermo, 90127 Palermo, Italy; andrea.cortegiani@unipa.it; 4 Department of Anesthesia, Intensive Care and Emergency, Policlinico Paolo Giaccone, University of Palermo, 90127 Palermo, Italy

High-flow nasal therapy (HFNT) was introduced into clinical practice in the early 2000s as a form of noninvasive respiratory support (NIRS). During the last 20 years, a growing body of evidence has shown the value of this intervention in the management of acute and chronic respiratory failure. Indeed, HFNT has transformed the landscape of research on and the clinical management of respiratory failure; according to a report released by NEJM in 2015 [1], it was judged as the most significant advancement in the field.

HFNT provides the delivery of humidified gas flow that meets or overcomes a patient’s peak inspiratory flow, assures a continuous washout of CO_2_ from anatomical dead space, generating a slight positive end-expiratory pressure effect that provides alveolar recruitment, and determines a reduction in inspiratory effort as well as an improvement in oxygenation with the delivery of a stable fraction of inspired oxygen that lowers the metabolic cost of ventilation [2]. HFNT is apparently easy to implement and requires minimal technical skill to set up, overcoming some of the major drawbacks of noninvasive ventilation (NIV), such as selecting the proper interface with which to minimize leaks and skin breakdown in addition to ventilator setting adjustments to mitigate patient–ventilator asynchronies; however, as with all forms of NIRS, HFNT should be used judiciously in the management of respiratory failure, in closely monitored patients and with well-trained staff to avoid potential harm as well as delayed intubation in acute settings [3], in selected patient populations in chronic settings, and it should not be considered a one-size-fits-all solution.

The beneficial effects of HFNT have mainly been demonstrated in patients with acute hypoxemic respiratory failure (AHRF), and clinical practice guidelines [4,5] recommend HFNT over conventional oxygen therapy (COT) as a first-line treatment in patients with de novo respiratory failure as it reduces intubation and the escalation of respiratory support, even if it does not affect mortality; however, the certainty of the evidence supporting the recommendation is moderate [4,5]; indeed, a systematic review and meta-analysis including immunocompromised patients with AHRF failed to identify any benefit of HFNT over COT [6]; therefore, heterogeneity under the umbrella of AHRF etiology among available studies should be taken into account. Indeed, many questions still remain open with regard to the use of HFNT for the right patient at the right time to manage AHRF. Furthermore, there is limited evidence on the optimal flow settings when using HFNT in different underlying diseases and how titrating this parameter might affect treatment failure or success [7] and patient comfort [8].

Despite the lack of evidence supporting the use of NIRS in pandemic viral illnesses [9], HFNT has been widely adopted for treating patients with COVID-19-related AHRF worldwide [10]. Multiple RCTs have been recently conducted in this patient population, but controversy still remains regarding HFNT’s efficacy and safety over COT. The HiFLo-COVID trial [11], conducted on patients with severe COVID-19-related AHRF, found that HFNT significantly reduced the risk of intubation and the time to clinical recovery compared to COT. Conversely, the COVID-HIGH trial [12], performed on patients with COVID-19-associated mild hypoxemia, revealed no clinical benefit of HFNT in reducing the likelihood of the escalation of respiratory support and clinical recovery. The RECOVERY-RS trial [13] showed no significant difference between HFNT vs. COT in the combined primary endpoint of tracheal intubation or mortality within 30 days. In contrast, the SOHO-COVID trial [14] showed lower intubation rates with HFNT but no effects on mortality compared to COT. Potential explanations for these differences in treatment effects are due to the heterogeneous severity of hypoxemia, inspiratory effort, timings of interventions, and types of outcomes assessed across trials, which were sometimes underpowered or prematurely terminated. A collaborative research effort is underway to synthesize the most important clinical outcomes from RCTs and will further elucidate the effects of HFNT in patients with COVID-19-related AHRF [15].

HFNT could also play a role in the management of hypercapnic respiratory failure in patients with acute exacerbation of COPD [16] (AECOPD), and it has also been shown to be beneficial in patients with AECOPD and coexisting bronchiectasis, improving gas exchange, dyspnea, and mucus production [17]. Moreover, HFNT was shown to be non-inferior to NIV in terms of PaCO_2_ reduction in mild-to-moderate AECOPD during a short-term follow-up [18]; however, a recent meta-analysis showed that the use of HFNT compared to NIV did not reduce the risk of mortality [19], highlighting the need for further research to assess long-term outcomes. In addition, HFNT has been proposed in AECOPD patients during breaks and weaning from NIV [20] as an alternative to COT, showing promising leads that should be followed-up in more depth.

The evidence for HFNT use in chronic respiratory failure has largely been focused on patients with COPD and bronchiectasis, suggesting that its use may influence exacerbations. HFNT prolonged the time to first exacerbation in a mixed population of COPD and bronchiectasis [21]. Another RCT on COPD patients showed a lower exacerbation rate than COT alone but no difference in hospitalizations [22]. In a more recent RCT on the same population, HFNT significantly reduced moderate-to-severe exacerbations without improving quality of life [23]. One case–control study in the current Special Issue showed a significant reduction in exacerbations and hospitalizations using HFNT in patients with bronchiectasis [24]. Taken together, these studies suggest that long-term HFNT might be effective in treating COPD and bronchiectasis “frequent exacerbator” phenotypes.

In the current era of personalized care, there is an exciting and bright future ahead of HFNT in both acute and chronic settings. The current Special Issue of the *Journal of Clinical Medicine* seeks to collect high-quality research to explore its further potential clinical applications and to strengthen the evidence of its current indications.
